# Revealing the Joint Mechanisms in Traditional Data Linked With Big Data

**DOI:** 10.1027/2151-2604/a000341

**Published:** 2019-02-22

**Authors:** Niek C. de Schipper, Katrijn Van Deun

**Affiliations:** ^1^ Department of Methodology and Statistics, Tilburg University, The Netherlands

**Keywords:** linked data, variable selection, component analysis, big data

## Abstract

**Abstract.** Recent technological advances have made it possible to study human behavior by linking novel types of data to more traditional types of psychological data, for example, linking psychological questionnaire data with genetic risk scores. Revealing the variables that are linked throughout these traditional and novel types of data gives crucial insight into the complex interplay between the multiple factors that determine human behavior, for example, the concerted action of genes and environment in the emergence of depression. Little or no theory is available on the link between such traditional and novel types of data, the latter usually consisting of a huge number of variables. The challenge is to select – in an automated way – those variables that are linked throughout the different blocks, and this eludes currently available methods for data analysis. To fill the methodological gap, we here present a novel data integration method.

In this era of big data, psychological researchers are faced with a situation where they can supplement the data they are accustomed to with novel kinds of data. For example, besides having questionnaire data also other types of data like experience sampling data, online behavior data, GPS coordinates, or genetic data may be available on the same subjects. Linking such additional blocks of information to the more traditional data holds promising prospects as it allows to study human behavior as the result of the concerted action of multiple influences. For example, having both questionnaire data on eating and health behavior together with data on genetic variants for the same subjects holds the key to finding how genes and environment act together in the emergence of eating disorders. Indeed, for most psycho-pathologies and many other behavioral outcomes, it holds that these are the result of a genetic susceptibility in combination with a risk provoking environment (Halldorsdottir & Binder, 2017). Thus, analyzing these traditional data together with novel types of data could provide us with crucial insights into the complex interplay between the multiple factors that determine human behavior.

Revealing the joint mechanisms in these integrated or linked data, such as the interplay between genes and environments, is challenging from a data analysis point of view because of the complex structure of the data. First, there is the novel kind of data that are very different from the traditional data we are used to work with: Instead of consisting of a limited number of targeted measurements, they consist of a huge amount of variables that have been collected without a specific focus. A typical example is so-called genome wide or “omics” data consisting of several thousands up to several millions of variables, but it is also the case with naturally occurring data like tweets, web page visits, or GPS signals (Paxton & Griffiths, 2017). As there is very little theoretical knowledge about the link between traditional and novel types of data, one is faced with a variable selection problem meaning that a data analysis method is needed that can reveal the relevant variables in an automated way. Such variable selection methods have been a very active research topic in statistics during the last years and led to approaches like lasso regression (Tibshirani, 1996) and sparse component analysis (Zou, Hastie, & Tibshirani, 2006). Second, the data consist of multiple blocks of data, and interest is in finding shared or joined mechanisms; this means revealing the sets of variables that are linked *throughout the blocks*. Current practice is to merge all data and apply methods developed for a single block of data, for example, state-of-the-art variable selection techniques such as lasso regression and sparse principal component analysis (PCA). This is an inappropriate approach that does not guarantee that variables from each of the blocks will be selected in case of joined mechanisms. First, usually the variables in the novel types of data outnumber those in the traditional data by far. Second, the blocks are dominated by specific information that is typical for the kind of processes they measure (e.g., behavioral processes and response tendencies in questionnaire data, biological processes in the genetic data) resulting in higher associations between the variables within blocks than between blocks. Hence, analyses that do not account for the multi-block structure of the data are highly unlikely to find the linked variables underlying the subtle joint mechanisms at play.

This paper proposes a novel data integration method that tries to overcome both of these challenges. It presents a significant extension of sparse PCA to the case of linked data, also called multi-block data. A simultaneous component approach (Kiers, 2000; Van Deun, Smilde, van der Werf, Kiers, & Van Mechelen, 2009) is taken, and proper constraints and regularization terms, including the lasso, are introduced to account for the presence of dominant block-specific sources of variation and to force variable selection.

The remainder of this paper is structured as follows: First, we will present the method as an extension of PCA to the multi-block case, and we will introduce an estimation procedure that is scalable to the setting of (very) large data. Second, using empirical data with three blocks of data on parent–child interactions, the substantive added value of singling out block-specific from common sources of variation and of sparse representations will be illustrated. Third, as a proof of concept, we will evaluate the performance of the method in a simulation study and compare it to the current practice of applying sparse PCA. We conclude with a discussion.

## Methods

In this section, first, the notation and data will be introduced; then the model, its estimation, model selection, and some related methods will be discussed.

### Notation and Description of Linked Data

In this paper, we will make use of the standardized notation proposed by Kiers (2000): Bold lower- and uppercases will denote vectors and matrices, respectively, superscript “*T*” denotes the transpose of a vector or matrix, and a running index will range from 1 to its uppercase letter (e.g., there is a total of *I* subjects where *i* runs from *i* = 1,…, *I*).

**Figure 1:**
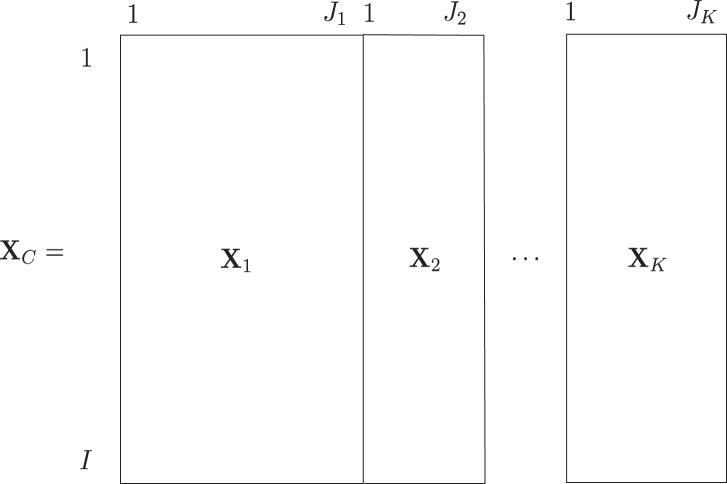
Example of a linked dataset: *K* data blocks are concatenated together where each data block **X**_*k*_ contains *J*_*k*_ variables for the same *I* subjects.

The data of interest are linked data, where for the same group of subjects, several blocks of data are analyzed together. A block of data is defined here as a group of variables that are homogeneous in the kind of information they measure (e.g., a set of items, a set of time points, a set of genes). Formally, we have *K* blocks of data **X**_*k*_ for *k* = 1, …, *K* with in each block scores of the same *I* subjects on the *J*_*k*_ variables making up the linked dataset (see Figure 1). Such data are called multi-block data (Tenenhaus & Tenenhaus, 2011) and are to be distinguished from multi-set data where scores are obtained on the same set of *J* variables but for different groups of subjects. Note that this paper is about multi-block data and does not apply to multi-set data. Furthermore, it is assumed that all data blocks consist of continues variables.

### Model Description of PCA and SCA

A powerful method for finding the sources of structural variation is principal component analysis (PCA; Jolliffe, 1986). Applied to a single block of data, PCA decomposes the data of an *I* × *J*_*k*_ data block **X**_*k*_ into,
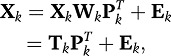
(1)where **W**_*k*_ denotes the *J*_*k*_ × *Q* component weight matrix, **P**_*k*_ denotes the *J*_*k*_ × *Q* loading matrix, and **E**_*k*_ denotes the error matrix. PCA is usually defined with 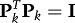 as identification constraint. In this formulation of PCA, the component scores are written explicitly as a linear combination of the variables. Let *t*_*iq*_ be the component score of subject *i* on a component *q*, then 
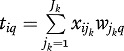
 which clearly shows that the component scores are a linear combination (weighted sum) of the variables scores. The PCA decomposition can also be applied to all **X**_*k*_ jointly by treating the multi-block data as one big matrix of ∑_*k*_|*J*_*k*_ variables,
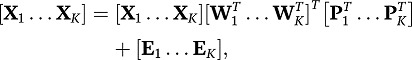
(2)or in shorthand notation,
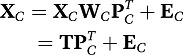
(3)


This model is the simultaneous component (SC) model (Kiers & ten Berge, 1989). An important property of SC models is that the same set of component scores underlies each of the data blocks: **X**_*k*_ = **TP**_*k*_ + **E**_*k*_ for all *k*. Note that these component scores are a linear combination of *all* the variables contained in the different blocks. Simultaneous components analysis (SCA) as defined in Equation (3) does not account for block-specific components nor does it imply variable selection. Therefore, we further extend it.

To account for the presence of block-specific components and to induce variable selection, we introduce particular constraints on the component weights **W**_*C*_ in the SC model; see model Equation (3). First, we will discuss the constraints to control for the presence of strong block-specific variation in the linked data, then we will discuss the sparseness constraints.

### Common and Distinctive Components

Consider the following example with two data blocks and three components with imposed blocks of zeroes,
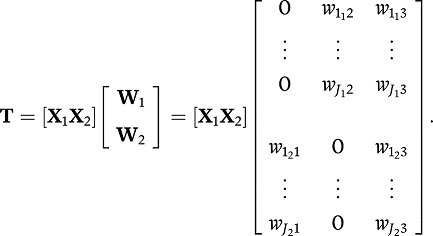
(4)(note that the variable subscripts in Equation 4 have their own subscript to denote the block they belong to; e.g., 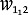 is the weight of the first variable in the *first block* on the second component while 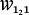 is the weight of the first variable in the *second block* on the first component). Due to the zero constraints, the scores on the first component only depend on the variables in the second block: 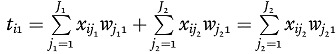. Likewise, the scores on the second component only depend on the variables in the first block. Because these components only incorporate the information of one particular type of data, we call them distinctive components as they reflect sources of variation that are particular for a block. These are examples of distinctive components that are formed by a linear combination of variables from *one* particular data block only. The third component **t**_3_ is a linear combination of the variables from both data blocks **X**_1_ and **X**_2_. Hence, it reflects sources of variation that play in both data blocks. We call these components common components. If there are more than three blocks the distinction between common and distinctive components can get blurred, for a detailed discussion see Smilde et al. (2017).

Usually, the most suitable common and distinctive structure for **W**_*C*_ given the data is not known. In the section on model selection below, we will discuss a strategy that can be used to find the most suitable common and distinctive weight structure for the data at hand.

### Sparse Common and Distinctive Components

The component weight matrix in Equation (4) has nonzero coefficients for all weights related to the common component and also for the nonzero blocks of the distinctive components. For the common component, for example, this implies that it is determined by all variables; no variable selection takes place. To accomplish variable selection, we impose sparseness constraints on the component weight matrix **W**_*C*_, in addition to the constraints that impose distinctiveness in Equation (4), for example,
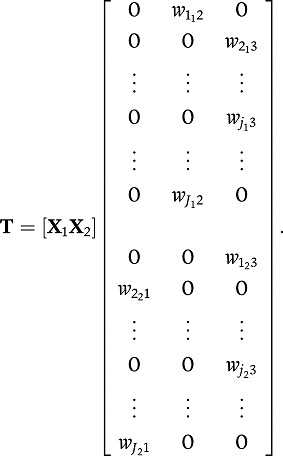
(5)


In this example, the common component is a linear combination of some instead of all variables; the same holds for the distinctive components. The number and position of the zeroes are assumed to be unknown. Next, we will introduce a statistical criterion that implies automated selection of the position of the zeroes. How to determine the number of zeroes, or the degree of sparsity, will be discussed in the section on model selection.

### Finding Sparse Common and Distinctive Components

To find the desired model structure with sparse common and distinctive components, the following optimization criterion is introduced:
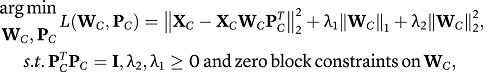
(6)with the notation 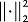 denoting the squared Frobenius norm, this is the sum of squared matrix elements, for example, 
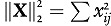
 and 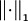 denoting the sum of the absolute values of the matrix elements, for example, 
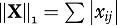
. The first term in the optimization criterion is the usual PCA least squares optimization criterion and implies a solution for **W**_*C*_ and **P**_*C*_ with minimal squared reconstruction error of the data by the components. The second and the third term are, respectively, the lasso and ridge penalty imposed on the component weight matrix **W**_*C*_. Both penalties encourage solutions with small weights; this is shrinkage toward zero (to minimize Eqation (6) not only a good fit is needed, but also weights that are as small as possible). The lasso has the additional property of setting weights exactly to zero (Tibshirani, 1996), introducing variable selection. The ridge penalty is needed in addition to the lasso penalty, because it leads to stabler estimates for **W**_*C*_ and eases the restriction that only *I* coefficients can be selected, which is the case when only the lasso penalty is used (Zou & Hastie, 2005). The tuning parameters λ_1_ and λ_2_ are the costs associated with the penalties, a larger value for the tuning parameter means that having large weights is more expensive, and thus imply more shrinkage of the weights or – in case of the lasso – also more zero component weights. The ridge and lasso regularization together with the common and distinctive component weight constraints can lead to the desired component weight estimates as outlined in Equation (5). Note that the function in Equation (6) also includes the special cases of PCA (when λ_1_ = 0 and λ_2_ = 0 and there are no constraints on **W**_*C*_) and of sparse PCA as presented by Zou et al. (2006) (when there are no constraints **W**_*C*_).

We call this novel approach of finding sparse common and distinctive components by minimizing Equation (6), SCaDS, short for: sparse common and distinctive SCA. In order to find the estimates **W**_*C*_ and **P**_*C*_ of SCaDS given a fixed number of components, values for λ_1_, λ_2_, and zero block constraints for **W**_*C*_, we make use of a numerical procedure that alternates between the estimation of **W**_*C*_ and **P**_*C*_ until the conditions for stopping have been met. Conditional on fixed values for **W**_*C*_, there is an analytic solution for **P**_*C*_, see, for example, ten Berge (1993) and Zou et al. (2006); for the conditional update of **W**_*C*_ given fixed values for **P**_*C*_, we use a coordinate descent procedure (see e.g., Friedman, Hastie, & Tibshirani, 2010). Our choice for coordinate descent is motivated by computational efficiency, meaning that it can be implemented in a way that it is a very fast procedure and scalable to the setting of thousands or even millions of variables without having to rely on specialized computing infrastructure. Another advantage is that constraints on the weights can be accommodated in a straightforward way because of the fact that each weight is updated in turn, conditional upon fixed values for the other weights; hence, weights that are constrained to have a set value are not updated. The derivation of the estimates for the component loadings and weights is detailed in Appendix A.

The alternating procedure results in a non-increasing sequence of loss values and converges^1^ to a fixed point, usually a local minimum. Multiple random starts can be used. The full SCaDS algorithm is presented in Appendix A, and its implementation in the statistical software R (R Core Team, 2016) is available from https://github.com/trbKnl/.

### Model Selection

SCaDS runs with fixed values for the number of components, their status (whether they are common or distinctive), and the value of the lasso and ridge tuning parameters. Often these are unknown and model selection procedures are needed to guide users of the method in the selection of proper values.

In the component and regression analysis literature, several model selection tools have been proposed. The scree plot, for example, is a popular tool to decide upon the number of components (Jolliffe, 1986) but also cross-validation has been proposed (Smilde, Bro, & Geladi, 2004). Given a known number of components, Schouteden, Van Deun, Pattyn, and Van Mechelen (2013) proposed an exhaustive strategy that relies upon an ad hoc criterion to decide upon the status (common or distinctive) of the components. Finally, tuning of the lasso and ridge penalties is usually based on cross-validation (Hastie, Tibshirani, & Friedman, 2009).

Here, we propose to use the following sequential strategy. First, the number of components is decided upon using cross-validation, more specifically the Eigenvector method. In a comparison of several cross-validation methods for determining the number of components, this method came out as the best choice in terms of accuracy and low computational cost; see Bro, Kjeldahl, Smilde, and Kiers (2008). Briefly, this method leaves out one or several samples and predicts the scores for each variable in turn based on a model that was obtained from the retained samples: For one up to a large number of components, the mean predicted residual sum of squares (MPRESS) is calculated and the model with the lowest MPRESS is retained. Second, a suitable common and distinctive structure for **W**_*C*_ is found using cross-validation: In this case, the MPRESS is calculated for all possible common and distinctive structures. Also in this case, we propose to use the Eigenvector method detailed in Bro et al. (2008). In a third and final step, the lasso and ridge parameters λ_1_ and λ_2_ are tuned using the Eigenvector cross-validation method on a grid of values, chosen such that overly sparse and non-sparse solutions are avoided.

An alternative to the sequential strategy proposed here is to use an exhaustive strategy in which all combinations of possible values for the components, their status, and λ_1_ and λ_2_ are assessed using cross-validation and retaining the solution with lowest MPRESS. However, there are known cases where sequential strategies outperform exhaustive strategies (Vervloet, Deun, den Noortgate, & Ceulemans, 2016), and, furthermore, sequential strategies have a computational advantage as the number of models that needs to be compared is much larger in the exhaustive setting. This number is already large in the sequential setting because all possible common and distinctive structures are inspected; these are in total 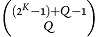 possible model structures.^2^ For example, with *K* = 2 data blocks and *Q* = 3 components, there are 
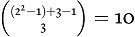
 possible common and distinctive structures to examine.

### Related Methods

The method introduced here builds further on extensions of principal component analysis. These include sparse PCA (Zou et al., 2006), simultaneous components with rotation to common and distinctive components (Schouteden et al., 2013), and sparse simultaneous component analysis (Gu & Van Deun, 2016; Van Deun, Wilderjans, van den Berg, Antoniadis, & Van Mechelen, 2011).

#### Sparse PCA

In practice, multi-block data are analyzed by treating them as a single block of variables. The problem of selecting the linked variables may then be addressed by using a sparse PCA technique. Zou et al. (2006) proposed a PCA method with a lasso and ridge penalty on the component weights. As previously discussed, this is a special case of the method we propose here (see Equation 6). The drawback of this approach is that it does not allow to control for dominant sources of variation.

#### SCA With Rotation to Common and Distinctive Components

Schouteden et al. (2013) proposed a rotation technique for multi-block data that rotates the components resulting from the simultaneous component analysis toward common and distinctive components: A target matrix is defined for the loading matrix that contains blocks of zeros for the distinctive components (similar to the model structure in Equation 4 and remains undefined for the remaining parts). In general, the rotated loadings will not be exactly equal to zero and may even be large. To decide whether the components are indeed common or distinctive after rotation, Schouteden et al. (2013) propose to inspect the proportion of variance accounted for (%VAF) by the components in each of the blocks: A component is considered distinctive when the %VAF is considerably higher in the block(s) underlying the component than in the other blocks; it is considered common when the %VAF is approximately the same in all blocks. This introduces some vagueness in defining the common and distinctive components. Furthermore, no variable selection is performed. An often used strategy in the interpretation of the loadings is to neglect small loadings. This corresponds to treating them as zeros and performing variable selection. As shown by Cadima and Jolliffe (1995), this is a suboptimal selection strategy in the sense that they account for less variation than optimally selected variables. At this point, we would also like to point out that the definition in terms of %VAF is not useful when the zero constraints are imposed on the component weights as the %VAF by a distinctive component can still be considerable for the block that does not make up the component. This is because the %VAF is determined by the component scores and *loadings* with zero weights not implying (near) zero loadings.

#### Sparse SCA

An extension of sparse PCA to the multi-block case was proposed by Van Deun et al. (2011). This approach allows for sparse estimation of the component weights using penalties that do not account for the multi-block structure like the ridge and lasso penalty but also using penalties that are structured at the level of the blocks like the group and elitist lasso (Kowalski & Torrésani, 2009; Yuan & Lin, 2006). The group lasso operates like the lasso at the block level, meaning that it sets whole blocks of coefficients equal to zero. The elitist lasso performs selection within each of the blocks, setting many but not all coefficients within each block equal to zero. Although sparse SCA allows for block-specific sparsity patterns, no distinction can be made between common and distinctive components because the penalties are defined at the level of the blocks (i.e., the same penalty for all components). Furthermore, the proposed algorithmic approach is not scalable to the setting of a (very) large number of variables: The procedure becomes slow and requires too much memory with a large number of variables.

#### SCA With Penalized Loadings

Recently, Gu and Van Deun (2016) developed an extension to sparse SCA by penalizing the loading matrix in a componentwise fashion, hence allowing for both common and distinctive components. The main distinguishing characteristic of this paper is that it penalizes the component weights and not the loadings. This raises the question whether this is very different, and if so, when to use penalized loadings and when to use penalized weights.

In regular unrotated PCA, loadings and weights are proportional or even exactly the same in approaches – such as the one taken here and by Zou et al. (2006) – that impose orthogonality on the matrix of weights or loadings (Smilde et al., 2004, p. 54). In case of penalties and sparsity constraints, however, loadings and weights take very different values and careful consideration should be given to their interpretation. Let us first consider the component weights. These are the regression weights in the calculation of the component scores and make the component scores directly observable. Sparseness of the component weights implies that the component scores are based on a selection of variables. An example, where such a weight-based approach may be most useful, is in the calculation of polygenic risk scores (Vassos et al., 2017). The loadings, on the other hand, measure the strength of association or correlation between the component and variable scores and give a more indirect or latent meaning to the components.

From an interpretational standpoint, there is also an important difference between the component weights and the component loadings. As ten Berge (1986) and references therein point out, the component weights convey how the components depend on the variables, whereas the component loading matrix conveys the relationship between the component and the variables. The component loadings can only be interpreted if the meaning of the components are more or less understood (if the components are not understood, you are inspecting the correlation between an observed item and something unknown, which is not insightful), in order to discover the meaning of the components, it is necessary to inspect the component weights first. To conclude, when the aim is to automatically detect the linked variables throughout different data blocks in order to reveal common mechanisms at play (e.g., a risk score based on genetic as well as environmental risk), in a situation where the components are not yet understood, sparseness of the weights is warranted.

Besides these differences in interpretation, there are also other differences between a sparse loading and a sparse weight approach. These include differences in reconstruction error, with the reconstruction error of a sparse loading approach being much larger, and differences in the algorithmic approach with algorithms for sparse weights being computationally more intensive and less stable than algorithms for sparse loadings.

## Empirical Data Examples

We will now provide two empirical data examples illustrating SCaDS. The purpose of these examples is twofold: one, to show how the analysis of linked data would go in practice when using SCaDS, and two, to showcase the interpretational gain of common and distinctive components for multi-block data and of sparseness in general.

### 500 Family Study

For the first data example, we will make use of the 500 Family Study (Schneider & Waite, 2008). This study contains questionnaire data from family members of families in the United States and aims to explore how work affects the lives and well-being of the members of a family. From this study, we will use combined scores of different items from questionnaires collected for the father, mother, and child of a family. These scores are about the mutual relations between parents, between parents and their child, and items about how the child perceives itself; see Table 1 for an overview of the variable labels. In this example, the units of observation are the families, and the three data blocks are formed by the variables collected from the father, the mother and the child. The father and the mother block both contain eight variables while the child block contains seven variables. There are 195 families in this selection of the data.

**Table 1:** Component weights for the family data resulting from SCA with Varimax rotation

	**w**_1_	**w**_2_	**w**_3_	**w**_4_	**w**_5_	**w**_6_
F: Relationship with partners	0.05	0.57	−0.02	0.03	−0.03	−0.09
F: Argue with partners	0.04	0.15	−0.03	−0.06	0.05	−0.47
F: Child’s bright future	−0.06	−0.08	0.15	0.47	0.01	−0.20
F: Activities with children	0.10	−0.03	0.04	−0.08	−0.63	−0.08
F: Feeling about parenting	−0.06	−0.15	0.06	0.06	−0.12	−0.40
F: Communication with children	−0.01	−0.01	−0.08	0.05	−0.49	−0.07
F: Argue with children	−0.11	−0.11	−0.06	−0.04	0.04	−0.53
F: Confidence about oneself	0.15	0.22	0.03	0.07	−0.08	−0.43
M: Relationship with partners	−0.07	0.60	0.06	0.01	0.06	0.03
M: Argue with partners	−0.27	0.16	−0.04	−0.26	0.06	−0.14
M: Child’s bright future	−0.38	−0.02	0.18	0.37	0.06	0.03
M: Activities with children	−0.27	−0.01	0.09	−0.10	−0.44	0.13
M: Feeling about parenting	−0.37	0.06	0.03	0.10	−0.01	−0.03
M: Communication with children	−0.42	−0.05	−0.03	−0.02	−0.16	0.05
M: Argue with children	−0.39	−0.14	−0.07	−0.15	0.17	−0.14
M: Confidence about oneself	−0.35	0.31	−0.07	−0.08	0.01	0.12
C: Self-confidence/esteem	−0.18	−0.10	−0.31	0.23	0.01	−0.01
C: Academic performance	−0.02	−0.03	−0.12	0.42	0.11	−0.04
C: Social life and extracurricular	0.08	0.12	0.01	0.37	−0.03	0.09
C: Importance of friendship	0.11	0.06	−0.37	0.23	−0.05	0.07
C: Self-image	−0.04	−0.02	−0.56	−0.07	0.01	−0.01
C: Happiness	0.02	−0.01	−0.55	−0.11	0.01	−0.04
C: Confidence about the future	−0.01	0.13	−0.19	0.27	−0.24	0.07

Variance accounted for (%)			55.2			
*Note*. The items starting with an F, M, or C belong to the father, mother, or child block, respectively.

In this section we will discuss the key steps in the analysis of linked data with SCaDS: pre-processing of the data, selecting the number of components, identifying the common and distinctive structure, the tuning of the ridge and lasso parameters, and the interpretation of the component weights.

#### Pre-Processing of the Data

In this example, the linked data blocks have been scaled and centered, meaning that all variables have a variance of one and a mean of zero. This is common practice in PCA and SCA and has been done to give all variables equal weight in the analysis. The blocks have not been individually weighted because they contain (almost) exactly the same number of variables.

#### Selecting the Number of Components

To find the number of components to retain, we made use of 10-fold cross-validation with the Eigenvector method. Figure 1 in Electronic Supplementary Material 1 (ESM 1) shows the MPRESS and the standard error of the MPRESS of the SC models with one up to ten components. The seven component solution is the solution with the lowest MPRESS; however, the solution with six components is within one standard error of the seven components solution. Relying on the one standard error rule, we will retain six components as this strikes a better balance between model fit and model complexity (Hastie et al., 2009).

#### Identifying the Common and Distinctive Structure

To find the common and distinctive structure of the component weights that fits best to the data, we performed 10-fold cross-validation with the Eigenvector method. Hence, we have six components and three data blocks, so there are a total of 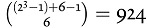 possible component weight structures to evaluate; the model with the lowest MPRESS was retained for further analysis; see Table 2. This is a model with one father-specific component (i.e., a component which is a linear combination of items from the father block only), one mother-specific component, one child-specific component, two parent (mother and father) components, and a common family component (a linear combination of items from all three blocks).

**Table 2:** The common and distinctive structure that resulted in the model with the lowest MPRESS out of the 924 possible models

	**w**_1_	**w**_2_	**w**_3_	**w**_4_	**w**_5_	**w**_6_
F: Relationship with partners	1	0	1	1	0	1
F: Argue with partners	1	0	1	1	0	1
F: Child’s bright future	1	0	1	1	0	1
F: Activities with children	1	0	1	1	0	1
F: Feeling about parenting	1	0	1	1	0	1
F: Communication with children	1	0	1	1	0	1
F: Argue with children	1	0	1	1	0	1
F: Confidence about oneself	1	0	1	1	0	1
M: Relationship with partners	0	1	1	1	0	1
M: Argue with partners	0	1	1	1	0	1
M: Child’s bright future	0	1	1	1	0	1
M: Activities with children	0	1	1	1	0	1
M: Feeling about parenting	0	1	1	1	0	1
M: Communication with children	0	1	1	1	0	1
M: Argue with children	0	1	1	1	0	1
M: Confidence about oneself	0	1	1	1	0	1
C: Self-confidence/esteem	0	0	0	0	1	1
C: Academic performance	0	0	0	0	1	1
C: Social life and extracurricular	0	0	0	0	1	1
C: Importance of friendship	0	0	0	0	1	1
C: Self-image	0	0	0	0	1	1
C: Happiness	0	0	0	0	1	1
C: Confidence about the future	0	0	0	0	1	1
*Notes*. The items starting with an F, M, or C belong to the father, mother, or child block, respectively. Zero indicates a component weight constrained to zero and one indicates a nonzero (free) component weight. The first component is a father component, the second component is a mother component, the third and the fourth are mother and father components, the fifth is a child component, and the sixth is a common component

#### Tuning of the Ridge and Lasso Parameters

**Figure 2:**
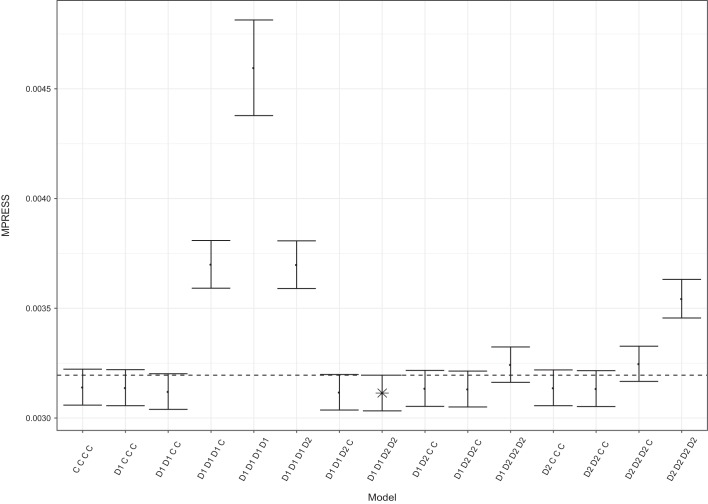
The MPRESS and standard error of all 15 models with different common and distinctive structures of the linked dataset from the ADNI study. Model “D1 D1 D2 D2” is the model with the lowest MPRESS. D1 denotes a distinctive component for the first block, D2 denotes a distinctive component for the second block, and C denotes a common component.

To further increase the interpretability of the components, we will estimate the component weights with the common and distinctive component weight structure resulting from the previous step but including sparseness constraints on the weights. This requires choosing values for the lasso and ridge tuning parameters λ_1_ and λ_2_. In this example, the solution is identified because we have more variables than cases; therefore we do not need the ridge penalty term; thus, the ridge penalty is set to 0. The optimal value for λ_1_ was picked by performing 10-fold cross-validation by the Eigenvector method for a sequence of λ_1_ values that results in going from no sparsity at all to very high sparsity in **W**_*C*_. The MPRESS and the standard error of the MPRESS of the models with the different values for the lasso parameter λ_1_ can be seen in Figure 2 in ESM 1; the one standard error rule was used to select the value for λ_1_.

#### Interpretation of the Component Weights

We subjected the data to a SCaDS analysis with six components, with zero constraints as in Table 2 and λ_1_ = 0.17. The estimated component weights are displayed in Table 3. For comparison, we also included component weights resulting from SCA followed by Varimax rotation in Table 1, and SCA followed by thresholding of the weights after rotation to the common and distinctive structure in Table 4. We will discuss the component weights from SCaDS first, after which we will compare these results to the alternative methods.

**Table 3:** Component weights for the family data as obtained with SCaDS

	**w**_1_	**w**_2_	**w**_3_	**w**_4_	**w**_5_	**w**_6_
F: Relationship with partners	0	0	0	0	0	0
F: Argue with partners	−0.57	0	0	0	0	0
F: Child’s bright future	0	0	0	0	0	0.56
F: Activities with children	0	0	0.61	0	0	0
F: Feeling about parenting	−0.12	0	0	0	0	0
F: Communication with children	0	0	0.39	0	0	0
F: Argue with children	−0.45	0	0	0	0	0
F: Confidence about oneself	−0.45	0	0	0	0	0
M: Relationship with partners	0	1.00	0	0	0	0
M: Argue with partners	0	0	0	−0.31	0	0
M: Child’s bright future	0	0	0	0	0	0.53
M: Activities with children	0	0	0.42	0	0	0
M: Feeling about parenting	0	0	0	−0.26	0	0.04
M: Communication with children	0	0	0	−0.44	0	0
M: Argue with children	0	0	0	−0.61	0	0
M: Confidence about oneself	0	0.26	0	−0.18	0	0
C: Self-confidence/esteem	0	0	0	0	−0.27	0.13
C: Academic performance	0	0	0	0	0	0.36
C: Social life and extracurricular	0	0	0	0	0	0.00
C: Importance of friendship	0	0	0	0	−0.41	0
C: Self-image	0	0	0	0	−0.56	0
C: Happiness	0	0	0	0	−0.45	0
C: Confidence about the future	0	0	0	0	−0.15	0.06

%VAF: per component	8.25	7.39	6.62	8.96	10.48	8.56
%VAF: total			50.3			
*Note.* The items starting with an F, M, or C belong to the father, mother, or child block, respectively.

**Table 4:** Component weights for the family data resulting from thresholded SCA with rotation to the common and distinctive structure

	**w**_1_	**w**_**2**_	**w**_3_	**w**_4_	**w**_5_	**w**_6_
F: Relationship with partners	0	0	−0.41	0.36	0	0
F: Argue with partners	−0.43	0	0	0	0	0
F: Child’s bright future	0	0	0	0	0	0.43
F: Activities with children	0	0	0.42	0.40	0	0
F: Feeling about parenting	−0.40	0	0	0	0	0
F: Communication with children	0	0	0	0	0	0
F: Argue with children	−0.45	0	0	−0.28	0	0
F: Confidence about oneself	−0.47	0	0	0	0	0
M: Relationship with partners	0	0	−0.46	0.33	0	0
M: Argue with partners	0	0	0	0	0	−0.26
M: Child’s bright future	0	0	0	0	0	0.38
M: Activities with children	0	0	0	0	0	0
M: Feeling about parenting	0	0	0	0	0	0
M: Communication with children	0	0.42	0	0	0	0
M: Argue with children	0	0	0	−0.31	0	0
M: Confidence about oneself	0	0.41	0	0	0	0
C: Self-confidence/esteem	0	0	0	0	−0.37	0
C: Academic performance	0	0	0	0	0	0.32
C: Social life and extracurricular	0	0	0	0	0	0.38
C: Importance of friendship	0	0	0	0	−0.43	0
C: Self-image	0	0	0	0	−0.50	−0.26
C: Happiness	0	0	0	0	−0.48	−0.29
C: Confidence about the future	0	0	0	0	−0.29	0

%VAF: per component	8.08	5.81	6.10	4.58	11.17	6.20
%VAF: total			41.9			
*Notes*. The items starting with an F, M, or C belong to the father, mother, or child block, respectively. Small absolute components weights have been set to zero in order to get just as much sparsity in the component weights as in the SCaDS solution in Table 3.

The six columns in Table 3 show the component weights obtained with SCaDS. In total, these components account for 50.3% of the variance. As imposed, the first component is father-specific, the second mother-specific, the third is a parent component, the fifth is child-specific, and the sixth component is a common family component. The fourth component was constrained to be a parent component but, as a result of the lasso penalty, became a second mother-specific component with nonzero loadings only from variables belonging to the mother block. Interestingly, the shared parent component is formed by the variables “activities with children,” “communication with children” of the father block, and “activities with children” of the mother block. The variable descriptions tell us that this component could be a parent–child involvement indicator. Large component weights for the common component are: “child’s bright future” in the mother and father block, and “self-confidence/esteem” and “academic performance” in the child block. This component indicates that a child’s self-confidence and academic performance is associated with both parents believing in a bright future for their child.

For comparison we included in Table 1 the component weights of the six components obtained using SCA with Varimax rotation, this is an unconstrained analysis with maximal VAF. In total, the six components explain 55.2% of the variance in the data; this is a bit more than the 50.3% obtained with SCaDS. Even this example with rather few variables is not straightforward to interpret because all variables contribute to each of the component. In this case, a more fair comparison is to rotate the component weights resulting from the SCA to the common and distinctive structure displayed in Table 2 and to threshold the small (in absolute value) coefficients as is often done in practice. We thresholded such that the same number of zero coefficients was obtained for each component as for SCaDS. The results of this analysis can be seen in Table 4. The first thing that strikes is that the variance accounted for drops to 41.9%. This confirms the observation made by (Cadima & Jolliffe, 1995) that the practice of thresholding is a flawed way to perform variable selection when the aim is to maximize the VAF. Also the meaning of the components changed, although the main patterns found in SCaDS can still be observed.

Concluding, these results illustrate well that identifying the common and distinctive structure in multi-block data eases the interpretation substantially, while still retaining a high variance accounted for.

An advantage of interpreting the component weights directly is that the researcher exactly knows the composition of the component. In some cases, the components themselves are used in subsequent analysis for example as predictors in a regression model. For the interpretation of that model, it is certainly useful to have a good grasp on what the predictors represent. Another advantage is that if the weights already have been estimated, then computing new component scores for new units of observation is straightforward. Because these component weights are sparse, only the items with nonzero component weights have to be measured to predict the component score of a new observed unit. This could greatly reduce the costs of predicting component scores for newly observed units.

### Alzheimer Study

For the second data example, we will use the Alzheimer’s Disease Neuroimaging Initiative (ADNI) data.^3^ The purpose of the ADNI study is “to validate biomarkers for use in Alzheimer’s disease clinical treatment trials” (Alzheimer’s Disease Neuroimaging Initiative, 2017).

The ADNI data is a collection of datasets from which we selected a dataset with items measuring neuropsychological constructs, and a dataset with gene expression data for genes related to Alzheimer’s disease. The neuropsychological data block consists of 12 variables containing items from a clinical dementia scale assessed by a professional and from a self-assessment scale relating to everyday’s cognition. The gene data block contains 388 genes. For a group of 175 participants, complete data for both the genetic and the neuropsychological variables is available. This is an example of a high-dimensional dataset where the number of variables exceeds the number of cases.

In this specific case, it would be interesting to see whether there is an association between particular Alzheimer-related genes and items from the clinical scales or whether the two types of data measure different sources of variation.

#### Pre-Processing of the Data

As in the previous example, the linked data blocks have been scaled and centered. Furthermore, as one block is much larger than the other, the blocks have been scaled to equal sum of squares by dividing each block by the square root of the number of variables in that block. In this way, the larger block does not dominate the analyses (see Van Deun et al., 2009, for a discussion of different weighting strategies).

#### Selecting the Number of Components

**Figure 3:**
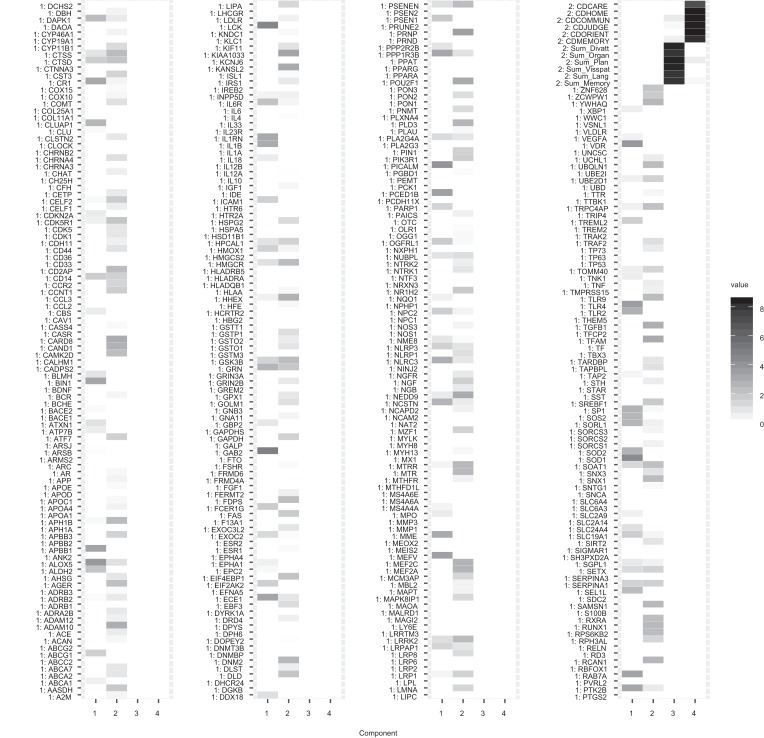
A heat plot of the absolute values of the component weights table of the final analysis for the ADNI data example. The variable names with prefix 1 denote variables belonging to gene expression block; names with prefix 2 denote variables belonging to the neuropsychological block. The figure has been broken row wise into four pieces to fit the page.

The number of components has been selected making use of 10-fold cross-validation with the Eigenvector method. This resulted in a four-component solution (see Figure 3 in ESM 1).

#### Tuning of the Ridge Parameter

**Figure 4:**
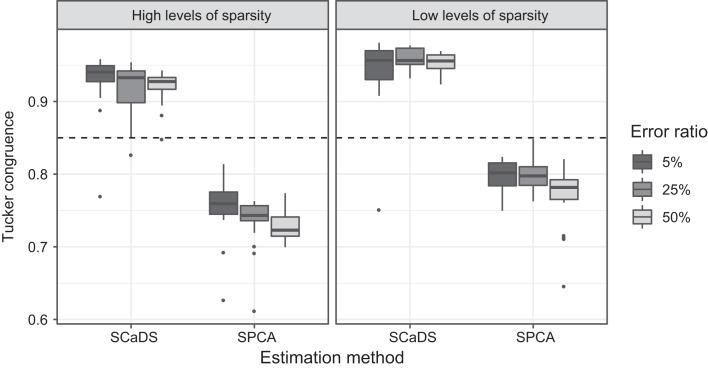
Tucker congruence coefficients between **W**_*C*_ and **Ŵ**_*C*_, where *I* = 100 and *J* = 500. Each condition is based on 20 replications; the dashed line indicates a Tucker congruence coefficient of 0.85.

This linked dataset contains more variables than cases; therefore, we included a ridge penalty (this is λ_2_ ≠ 0) to make the solution stable. To tune the value of the ridge parameter, we performed 10-fold cross-validation with the Eigenvector method on a sequence of values. The resulting MPRESS statistics and standard errors thereof are shown in Figure 4 in ESM 1. The value within one standard error of the lowest MPRESS was retained for further analyses.

#### Identifying the Common and Distinctive Structure

To find the common and distinctive structure of the component weights which fits best to the data, we performed 10-fold cross-validation with the Eigenvector method on all possible structures. In this example, we have four components and two data blocks, so there are a total of 
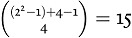
 possible component weight structures to evaluate. After cross-validation we found the model with the lowest MPRESS to be a model with four distinctive components: two for each block; see Figure 2 for the MPRESS and standard error of the MPRESS of all the 15 models.

#### Tuning of the Lasso Parameters

**Figure 5:**
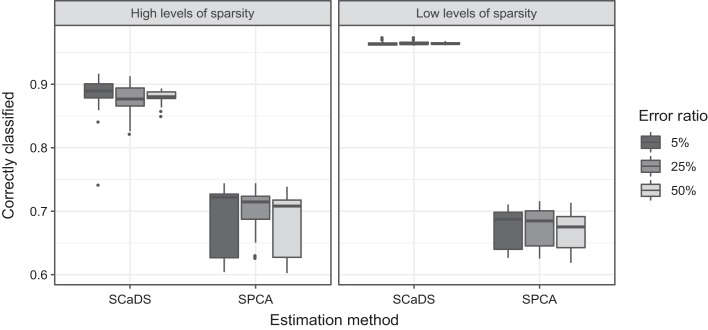
Percentage of correctly classified zero and nonzero weights between **W**_*C*_ and **Ŵ**_*C*_, where *I* = 100 and *J* = 500. Each condition is based on 20 replications.

A final step in selecting a suitable model for the ADNI data is the tuning of the lasso parameter to obtain sparsity in the component weights beyond the zeroes resulting from the imposed common and distinctive structure. The value of the lasso parameter was determined with 10-fold cross-validation (Eigenvector method). The MPRESS of the models for different values of the lasso parameter can be seen in Figure 5 in ESM 1; the largest value of λ_1_ within one standard error of the lowest MPRESS was retained for the final SCaDS analysis.

#### Interpretation of the Component Weights

The component weights of the final analysis with the chosen meta-parameters are summarized in a heat plot in Figure 3. The first two components contain only items from the gene expression block, and the third and the fourth component only contain items from the neuropsychological data block. Notably, the third component mainly contains items of the self-assessment scale while the fourth component mainly contains items of the dementia scale assessed by the clinician.

Concluding, this particular example shows that SCaDS can also be applied in the setting of (many) more variables than observation units. Whether the obtained results also make sense from a neuropsychological perspective needs further investigation.

## Simulation Studies

We tested the performance of SCaDS in finding back a sparse common and distinctive model structure in a controlled setting using simulated data. First of all, we were interested to see whether accounting for the presence of block-specific components in **W**_*C*_ would result in improved estimates compared to a sparse PCA analysis of the concatenated data. If there is no improvement of the estimated weights by SCaDS over sparse PCA, sparse PCA can be used for the analysis of multi-block data and there is no need for SCaDS. Second, we tested the performance of the cross-validation method in finding back the right common-distinctive structure given the correct number of components.

### Recovery of the Model Parameters Under the Correct Model

The data in the first simulation study were generated under a sparse SCA model with two data blocks and three components, of which one component is common and two are distinctive (one distinctive for each data block; see Equation 5 for such a model structure). The size of the two data blocks was fixed to 100 rows (subjects) and 250 columns (variables) per block.

We generated data under six conditions, resulting from a fully crossed design determined by two factors. A first factor was the amount of noise in the generated data with three levels: 5%, 25%, and 50% of the total variation. The second factor was the amount of sparsity in **W**_*C*_ with two levels: a high amount of sparsity (60% in all three components) and almost no sparsity (2% in the common component and 52% in the distinctive components) in the component weight matrix **W**_*C*_. In each condition, 20 datasets were generated. We refer the reader to Appendix B for the details on the procedure we used to generate data with the desired model structure.

All datasets were analyzed using both the SCaDS method introduced here and the sparse PCA analysis introduced by Zou et al. (2006) and implemented in the elastic net R package (Zou & Hastie, 2012). SCaDS was applied with correct input for the zero block constraints on the component weight matrix, this is with input of the common and distinctive structure that underlies the data. Sparse PCA was applied with input of the correct number of zero component weights in **W**_*C*_ and this for each component (sparse PCA can be tuned to yield exactly a given number of zero coefficients because it relies on a LARS estimation procedure; Tibshirani, Johnstone, Hastie, & Efron, 2004). Using sparse PCA with the correct number of zero component weights is equal to supplying the analysis with a perfectly tuned lasso parameter. In order to achieve a perfectly tuned lasso parameter for SCaDS, we used an iterative scheme based on the bisection method for root finding. The method boils down to estimating the model with a certain lasso value, after which depending on the number of nonzero weights in **Ŵ**_*C*_ compared the number of nonzero weights in **W**_*C*_, the lasso is increased or decreased. This process is repeated until the number of nonzero component weights in **Ŵ**_*C*_ is within 0.01% of the number of nonzero component weights in **W**_*C*_. The ridge parameter λ_2_ was tuned for one particular dataset in each of the six conditions with cross-validation and picked according to the one standard error rule. (The ridge was not tuned for each individual dataset because of computational constraints.).

In order to quantify how well the component weight matrix **W**_*C*_ can be recovered by SCaDS and sparse PCA of the concatenated data, we calculated Tucker’s coefficient of congruence between the model structure **W**_*C*_ and its estimate **Ŵ**_*C*_ as resulting from SCaDS and sparse PCA. Tucker’s coefficient of congruence (Lorenzo-Seva & ten Berge, 2006) is a standardized measure of proportionality between two vectors, calculated as the cosine of the angle between two vectors. Note that **W**_*C*_ and **Ŵ**_*C*_ are vectorized first before they are compared. A Tucker congruence coefficient in the range .85–.95 corresponds to fair similarity between vectors, while a Tucker congruence coefficient of > .95 correspond to near equal vectors (Lorenzo-Seva & ten Berge, 2006). Furthermore, we also calculated the percentage of correctly as (non-)zero classified component weights.

Box plots of Tucker’s coefficient of congruence between **W**_*C*_ and **Ŵ**_*C*_ are shown in Figure 4, both for the estimates obtained with our SCaDS method and with sparse PCA. The two panels correspond to the two levels of sparseness; within panels, the box plots differ with respect to the method used to estimate the weight matrix and the noise level. In all conditions, SCaDS has on average higher congruence than sparse PCA. This indicates that controlling for block-specific sources of variation results in a better recovery of the model coefficients (given the correct model). Furthermore, the bulk of Tucker congruence coefficients obtained when using SCaDS are above the threshold value of 0.85 thus indicating fair similarity of the estimated component weights to the model component weights. Sparse PCA, on the other hand, has almost all solutions below the 0.85 threshold. The manipulated noise and sparseness factors had some influence on the size of Tucker’s congruence. First, as one may expect, congruence decreased with an increasing level of noise. Second, comparing the left panel (high level of sparsity) to the right panel (low level of sparsity), Tucker congruence was higher for the low level of sparsity.

The box plots in Figure 5 show the percentage of correctly classified component weights for both estimation procedures in each of the six conditions. An estimated component weight is counted as correctly classified if it has nonzero status in **W**_*C*_ as well as in **Ŵ**_*C*_ or if it has zero status in **W**_*C*_ as well as in **Ŵ**_*C*_. Not surprisingly, SCaDS does far better compared to sparse PCA, this because SCaDS makes use of true underlying structure of the data. More importantly, these results show that if the data do actually contain an underlying multi-block structure, sparse PCA is not able to find this structure by default, too much weights are incorrectly classified. For good recovery of the component weights, it necessary to take the correct block structure into account.

Concluding, this simulation study shows that a multi-block structure is not picked up by sparse PCA by default. Furthermore, the simulation results show that to have satisfactory component weights estimates the correct multi-block structure needs to be taken into account. In practice, the underlying multi-block structure of the data is unknown. Hence, model selection tools that can recover the correct model are needed.

### Finding the Underlying Common and Distinctive Structure of the Data

In the previous section, we concluded that in order to have good estimation, the correct underlying multi-block structure needs to be known. In this section, we will explore to what extent 10-fold cross-validation with the Eigenvector method can be used to identify the correct underlying block structure of the data, assuming the number of components is known. We will consider both a high- and a low-dimensional setting.

In the high-dimensional setting, data were generated under the same conditions as the previous simulation study but analyzed without input of the correct common-distinctive model structure. Instead, for each of the generated datasets, we calculated the MPRESS and its standard error for all possible combinations of common and distinctive components; this is 10 possible models for each generated dataset (2 data blocks 3 combinations). The models are estimated without a lasso penalty (this is λ_1_ = 0) and with the same value for the ridge parameter as in the previous simulation study.

**Figure 6:**
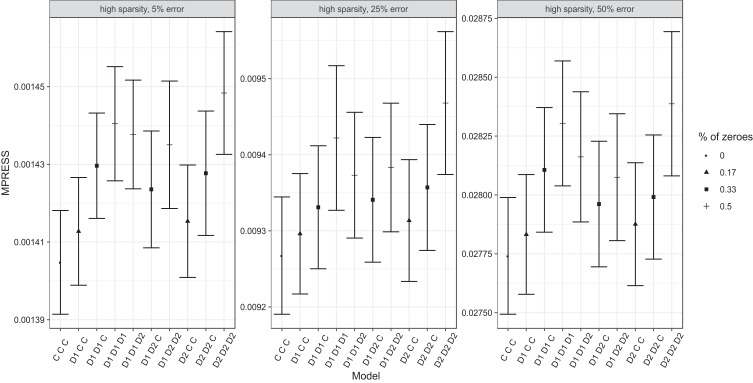
The MPRESS and standard error of the MPRESS of all common and distinctive structures in three conditions. Percentage (%) of zeroes refers to the amount of zeroes in the common and distinctive structure. “D1 D2 C” is the data generating structure. D1 denotes a distinctive component for the first block, D2 denotes a distinctive component for the second block, and C denotes a common component.

We illustrate the results obtained for the first three generated datasets in the high sparsity condition in Figure 6. The correct model used to generate the data is the model labeled “D1 D2 C” (representing a model with one distinctive component for each block and one common component). The plots show that the most complex model (this is the unconstrained “C C C” model) always has the lowest MPRESS. Furthermore, the plots show that model fit decreases for models with more imposed zeroes. This means that 10-fold cross-validation with the Eigenvector method favors models that are overfitted (i.e., models with too many nonzero coefficients). To remedy this situation, the one standard error rule has been proposed (Hastie et al., 2009). Here, this means that the model with the lowest complexity (or, the highest number of zeroes) is chosen that still falls within one standard error of the model with the lowest MPRESS; if this is more than one model, the model with lowest MPRESS is chosen. The results in Figure 6 suggest that this may lead to the correct model in a number of cases (the two panels at the right).

The results of the full simulation study are summarized in Table 5. The column labeled “Best model” shows the proportion of cases where the true model was selected based on choosing the model with lowest MPRESS. This strategy never results in selecting the correct model. Upon closer inspection of the results (e.g., Figure 6), the model with lowest MPRESS often was the unconstrained model. Whether the correct model would be selected when applying the one standard error rule (i.e., choosing the model with the highest MPRESS but within one standard error of the model with the lowest MPRESS) can be seen in the column labeled “One Std Error rule.” Unfortunately, this does not seem to be the case very often, in only about 10% of the cases the correct model was chosen based on this heuristic. Clearly, cross-validation as a method for selecting the true common-distinctive model structure does not work in the high-dimensional setting.

**Table 5:** Results of the simulation study for finding the underlying common and distinctive structure with 10-fold cross-validation in the high-dimensional setting

Sparsity	Noise (%)	Best model^a^	One standard error rule^b^
High	5	0	0.05
High	25	0	0.35
High	50	0	0.15
Low	5	0	0.20
Low	25	0	0.05
low	50	0	0.05
*Notes.*^a^The proportion of cases where the model with the true structure is the model with the lowest MPRESS. ^b^The proportion of cases the model with the true structure is selected based on the one standard error rule. The results are based on 20 replications in each condition.

**Figure 7:**
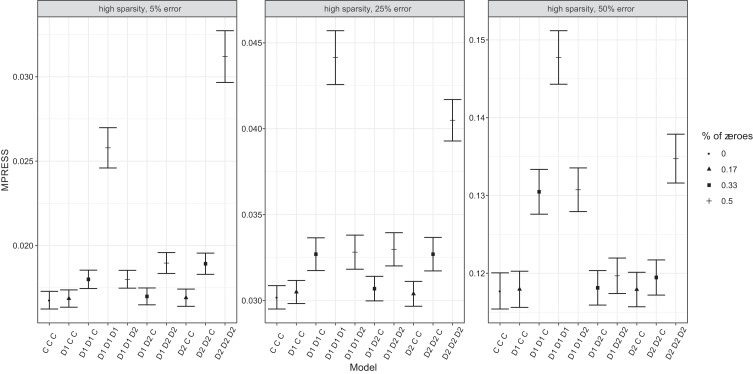
The MPRESS and standard error of the MPRESS of all common and distinctive structures in three conditions. Percentage (%) of zeroes refers to the amount of zeroes in the common and distinctive structure. “D1 D2 C” is the data generating structure. D1 denotes a distinctive component for the first block, D2 denotes a distinctive component for the second block, and C denotes a common component.

We also included results of a second simulation study to see how 10-fold cross-validation would perform in the low-dimensional case: data were generated as previously but with 195 cases and 20 variables. Figure 7 includes results of three specific generated datasets. Still cross-validation based on selecting the model with the lowest MPRESS is biased toward more complex models with fewer zero constraints. However, using the one standard error rule, often the correct model is selected. A full summary of the results can be seen in Table 6.

**Table 6:** Results of the simulation study for finding the underlying common and distinctive structure with 10-fold cross-validation in the low-dimensional setting

Sparsity	Noise (%)	Best model^a^	One standard error rule^b^
High	5	0	0.60
High	25	0	0.95
High	50	0	0.85
Low	5	0	0.65
Low	25	0	1.00
Low	50	0	0.85
*Notes.*^a^The proportion of cases where the model with the true structure is the model with the lowest MPRESS. ^b^The proportion of cases the model with the true structure is selected based on the one standard error rule. The results are based on 20 replications in each condition.

Concluding, 10-fold cross-validation with the Eigenvector method and using the one standard error rule does seem to work for selecting the correct common-distinctive model structure in the low-dimensional setting. However, in the high-dimensional setting overly complex models are chosen even when using the one standard error rule. Clearly, other model selection tools have to be tested, also taking into account that here only one model selection step was isolated. Although we suggested a sequential strategy to reduce the computational burden, simultaneous strategies may be needed in order to find the correct model.

## Discussion

In this era of big data, researchers in psychology often have novel types of data available to supplement the more traditional types of data they are accustomed to. This opens the avenue to a more informed understanding of the human behavior system; the different types of data usually probe different components of the behavioral system and by integrating them a more complete view is obtained. To get this deeper understanding that goes beyond a fragmented view, it is crucial that the following questions can be answered: How do the components of the human behavior system interact and what do they contribute independently from the other components? As we argued in this paper, this means disentangling joint sources of variation from specific sources of variation present in the data. A further complicating factor in the analysis of linked traditional and novel data resides in the often untargeted collection of the novel data: This not only leads to a very large number of variables but also to the collection of variables that may or may not be relevant for the problem under study. On the side of data analysis, this requires methods that are computationally efficient and capable of automated variable selection. To address these issues, we introduced SCaDS, a novel variable selection method that is suitable to detect the common and specific mechanisms at play. In this paper, we proposed the SCaDS model, a procedure to estimate the model parameters and an implementation of the algorithm in the freely available statistical software R. Importantly, the proposed implementation of SCaDS can handle a large number of variables including cases where the total number of variables exceeds the number of observations.

We illustrated how to use SCaDS using publicly available data from the 500 Family Study (Schneider & Waite, 2008) using a block of data for father, for mother, and for their child. The interpretational advantage of using a sparse common and distinctive model structure was clearly shown. We also included an application to Alzheimer patient data including a block with genetic variables and a block with cognitive scale variables to illustrate the use of SCaDS in the high-dimensional setting. Furthermore, support for the superior performance of SCaDS compared to sparse PCA of the concatenated data in estimating back the model parameters was convincingly shown in a simulation study. Especially in situations where the number of variables was large compared to the number of observation units, SCaDS outperformed the approach of applying sparse methods for a single block of data while ignoring the multi-block structure.

In this paper, we used cross-validation as a tool to determine the meta-parameters of the SCaDS method, namely the number of common and distinctive components and the level of sparsity. For data generated in the low-dimensional setting, satisfactory results were obtained, yet, in the high-dimensional setting, we observed a bias toward overly complex models. More research needs to be done – including the use of simulation studies – to investigate if cross-validation indeed recovers the correct number of common and distinctive components and the degree of sparseness. Other alternatives that have been proposed in the literature need to be explored as well, including the convex hull method (Timmerman, Kiers, & Ceulemans, 2016; Wilderjans, Ceulemans, & Meers, 2012). Of particular interests are model selection tools that are less computationally intensive than cross-validation like the index of sparseness (Trendafilov, 2013).

To conclude, SCaDS is a promising method for the analysis of multi-block data that yields insightful representations of linked data: Intricate relations between very different sources of information on human behavior are revealed, even in presence of irrelevant variables. Here, the methodology was introduced and showcased on data with a relatively modest number of variables. The implementation proposed here is scalable to the high-dimensional case of very large sets of variables but more work is needed to study the performance of SCaDS in such settings using both simulated and empirical data.

## Electronic Supplementary Material

The electronic supplementary material is available with the online version of the article at https://doi.org/10.1027/2151-2604/a000341 *ESM 1.* Figures (.pdf)
MPRESS and standard errors of the models estimated with different numbers of components and parameter values.

## Footnotes

^1^ Under mild conditions that hold in practice. An example where there is no convergence is starting from **W**_*C*_ = **0**.

^2^ The number of possible common and distinctive structures for a single component weight vector is equal to 2^*K*^ − 1, because each of the *K* data block segments can either be constrained to be equal to zero or can be unconstrained; the case where the component is constrained to be equal to zero everywhere is not considered, hence the minus 1. For each of the *Q* components, one of these structures can be picked (with replacement meaning that two components can be of the same type, e.g., two common components) where the order of the components is of no importance.

^3^ The ADNI was launched in 2003 as a public–private partnership, led by Principal Investigator Michael W. Weiner, MD. The primary goal of ADNI has been to test whether serial magnetic resonance imaging (MRI), positron emission tomography (PET), other biological markers, and clinical and neuropsychological assessment can be combined to measure the progression of mild cognitive impairment (MCI) and early Alzheimer’s disease (AD). For up-to-date information, see http://www.adni-info.org.

## Appendix A

### Description Algorithm

In order to obtain a solution for **W**_*C*_ with SCaDS, the following objective function needs to be minimized given selected values for the tuning parameters: λ_2_, λ_1_, the number of components and a common-distinctive component weight structure,

(9)

where , ,  and *I*, *J*_*k*_, and *Q* denote the number of cases, variables in block *k* and components, respectively. For ease of notation, we will drop the subscript *C* and let Σ_*k*_|*J*_*k*_ = *L* where *l* = 1, …, *L*.

A solution to Equation (9) can be obtained using an alternating least squares approach. Meaning estimates for **P** can be obtained by minimizing (9) conditional on **W** and vice versa. The alternating least squares minimization is repeated until the convergence criterion has been reached. First, we will discuss the optimizing Equation (9) with respect to **P** conditional on **W**, then we will discuss the optimization of **W** conditional on **P**.

The loading matrix **P** which minimizes Equation (9) conditional on **W** has an analytic solution given **P**^*T*^**P** = **I**. The solution is given by **P** = **UV**^*T*^, where **U** and **V** are the left and right singular vectors of **X**^*T*^**XW** (ten Berge 1993; Zou et al., 2006).

In order to obtain the component weight matrix **W** that minimizes Equation (9) conditional on **P**, we implemented a coordinate descent optimization procedure. In order to use coordinate descent to get estimates for **W**, we rewrite Equation (9) as follows,

(10)

where  denotes the Kronecker product, between the factor loading matrix **P** and the data **X** and where *vec*(**X**) denotes the column vector representation of **X**. In the Kronecker product each entry of **P** is multiplied with **X** and put together in one big matrix. An example of (**P** ⊗ **X**) and *vec*(**X**) is given by:

In this optimization problem, an elastic net regression problem can be recognized (Zou & Hastie, 2005), to demonstrate this let *vec*(**X**) be the outcome variable **y**, let  be the matrix **X*** and let *vec*(**W**) be the coefficient vector **β**. For clarity, we index the rows and columns of **X*** by *m* and *n*, respectively. To find estimates for **β**, we will use a coordinate descent procedure (Friedman et al., 2010). This is an iterative procedure for which the solution to each successive approximation of β_*n*_ is given by,

(11)

where *S*(*z,* γ) denotes the soft-thresholding operator: sign(*z*)(|*z*| − γ)_+_ and (α)_+_ denotes the positive part function,  denotes the current estimate of β_*n*_, and *r*_*m*_ denotes the current residual . The coefficients get updated until a convergence criterion has been reached. The component weights constraint by the common and distinctive structure are skipped by (9) and therefore stay zero. A minimum of (9) can be found by successively alternating between the estimation of **P** and **W** until the convergence criterion has been reached. Note that this optimization problem is not convex, meaning that a minimum of (9) does not have to be the global minimum. Note that **P** conditional on **W** and vice versa have global optima because they result from solving convex optimization problems.

The coordinate descent procedure in (9) in its current form is infeasible to work with and has to be rewritten in order for it to be usable in practice. This has two reasons, first, the procedure relies on **X***, **X*** can get very large which may result into memory problems and makes the procedure slow. Second, **r** needs to be calculated every time β_*n*_ has been updated; this is costly as **r** = **y** − **X*****β**. In order to make the coordinate descent procedure efficient, the Kronecker product needs to be avoided. This can be done by noting some properties of . First, let us focus on the dot product of two columns of **X*** where each entry in those columns is multiplied by p_•q_ carrying the same index *q*, (index numbers with a “•” follow from the context) then because of the orthonormality of **P** recognize that  can be rewritten as follows,

(12)

This means that the dot product of two columns of **X*** multiplied by the same *p*_*•q*_, is the same as the dot product of the corresponding columns from the original data. The inner product of two columns from **X*** multiplied by *p*_*•q*_ and *p*_*•z*_ where *q* ≠ *z* results in . Second, recognize that in Equation (11),

(13)

Making use of Equations (12) and (13), the updating Equation (11) can be rewritten as,

(14)

which does not rely on the Kronecker product. Note that each time a coefficient gets updated, the vector  needs to be calculated again; this matrix vector product can be partially avoided by calculating  before the updating of the weights in **w**_*q*_ starts. If during the updating *w*_*lq*_ is put to zero, the vector  gets added back to , and if *w*_*lq*_ gets updated to a new value, the difference  is added back to . The coordinate descent algorithm is given by Algorithm (1), and the full SCaDS algorithm is given by Algorithm (2).

#### Algorithm 1: Coordinate Descent Algorithm for the Component Weights

1: **procedure** CoorDeS(**X, W, P**, λ_2_, λ_1_, ϵ, fixed structure for **W)**

2:  **c** = empty array of length *I*

3:  **while** convergence criterion ϵ is not satisfied **do**

4:   **for** *q* = 1 to *Q* **do**

5:    

6:    **for** *l* = 1 to *L* **do**

7:    **if** *w*_*lq*_ is not constraint to 0 **then**

8:    

9:    

10: *b* = sign(*a*)(|*a*| − λ_1_)_+_

11:    

12:     **if** |*a*| < *λ*_1_**then**

13:    *w*_*jq*_ = 0

14:    

15:    **else**

16:    

17:    **end if**

18:  **end if**

19:  **end for**

20: **end for**

21: **end while**

22: **return W**

23: **end procedure**

#### Algorithm 2: Algorithm for SCaDS

1: **procedure** SCaDS**(X**, *Q*, *λ*_2_, λ_1_, ϵ_1_, ϵ_2_, fixed structure for **W)**

2: initialize

3: **while** convergence criterion ϵ_1_ is not satisfied **do**

4:  store **U**, **V**^*T*^ from SVD(**X**^*T*^**XW**)

5:  **P = UV**^*T*^

6:  **W =** CoorDes(**X, W, P**, λ_2_, λ_1_, ϵ_2_, fixed structure for **W)**

7: **end while**

8: **return W, P**

9: **end procedure**

## Appendix B

### Specifics Simulation Study

As a starting point for the generation of data in the simulation study, initial data matrices were generated according to , where  for *i* = 1, …, 100 subjects. The variables in the resulting dataset **X**^(1)^ were standardized to have zero mean and unit variance. Let **UDV**^T^ be the singular value decomposition of the standardized matrix **X**^(2)^. Then, the standard PCA decomposition can be obtained by setting the loading matrix **P**^(1)^ equal to the first three columns of **V** and setting **W**^(1)^ also equal to the first three columns of **V** (note that the basis PCA model with orthogonality of the loadings indeed has equal weights and loadings). As a next step, we imposed the common and distinctive sparse structure on **W**^(1)^ as follows: First, a non-sparse common and distinctive structure was obtained by setting – for the distinctive components – all those component weights that correspond to the variables of a particular block equal to zero (see Equation 4); next, sparseness of the common component and of the nonzero parts of the distinctive component was imposed by setting all coefficients with an absolute value lower than some threshold to zero. The threshold was taken such that the level of sparseness defined by the condition was attained. The resulting matrix of component weights W is the model structure. Subsequently, the model structure for P was obtained by setting the loadings equal to solution of the least squares problem  conditional on **W** see Appendix A. Finally, the data **X*** that were used as the input to the SCaDS and sparse PCA analyses were obtained by adding noise,

(7)

where **E** is a random error matrix where the rows are generated from , and where *c* is a scalar that controls the signal to noise ratio (SNR) in **X***. The SNR is calculated as follows.

(8)

To obtain the scalar *c* to get the desired SNR, substitute the SNR (in the simulation study these 0.05, 0.25, 0.5) into Equation (8) and solve for *c*. No multiple starts were used in the simulation study; the algorithm was started with a “warm” start by initializing **W** with first three columns of **V** from **X*** = **UDV**^T^.
