# Supplementary material for: Revealing the Joint Mechanisms in Traditional Data Linked With Big Data
Source: Z Psychol. 2019 Feb 22;226(4):212–31. doi: 10.1027/2151-2604/a000341 (PMC6736194; doi:10.1027/2151-2604/a000341)
Supplement: Supplementary file 1 [file zfp_226_4_212_esm1.pdf]

Electronic Supplementary Material for

de Schipper, N. C., & Van Deun, K. (2018). Revealing the Joint Mechanisms in Traditional Data Linked With Big Data. *Zeitschrift für Psychologie*. https://doi.org/10.1027/2151-2604/a000341

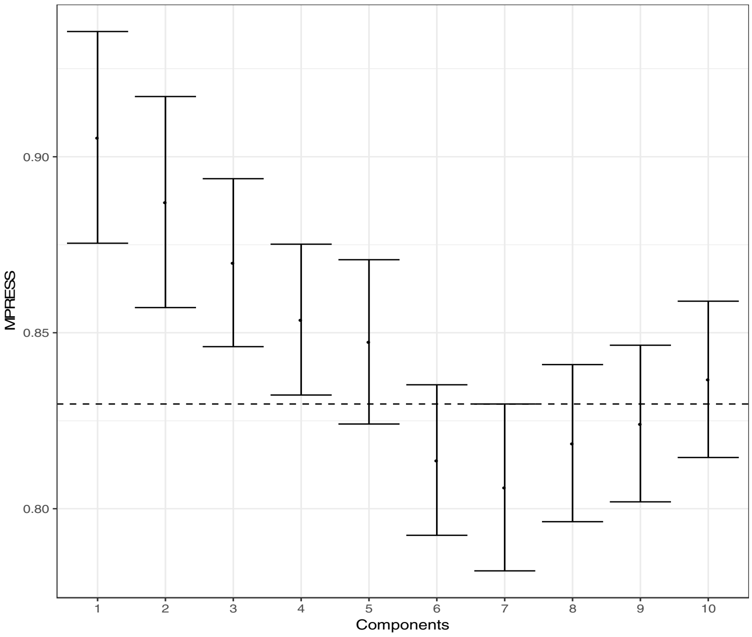

Figure 1. The MPRESS and standard error of the models estimated with different numbers of components. The model estimated with seven components was the model with the lowest MPRESS, the model with six components was chosen for the final analysis.

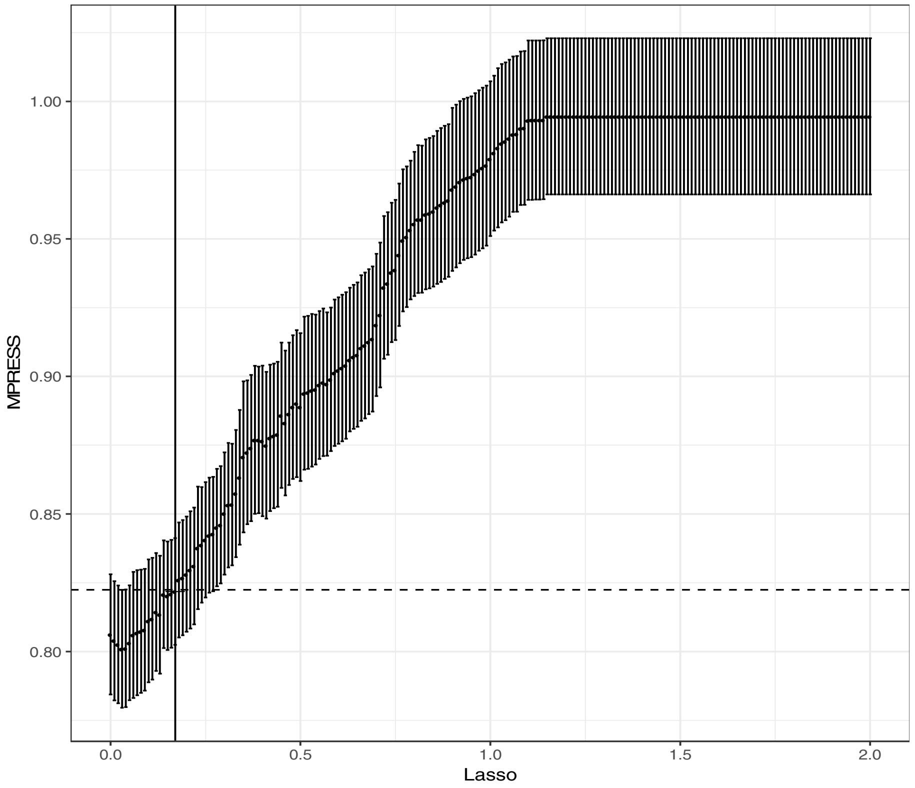

Figure 2. The MPRESS and standard error of the models for different values of the lasso parameter estimated with six components. The models below the dashed line are all models within one standard error of the model with the lowest MPRESS. The solid line indicates the lasso value that was picked for further analysis.

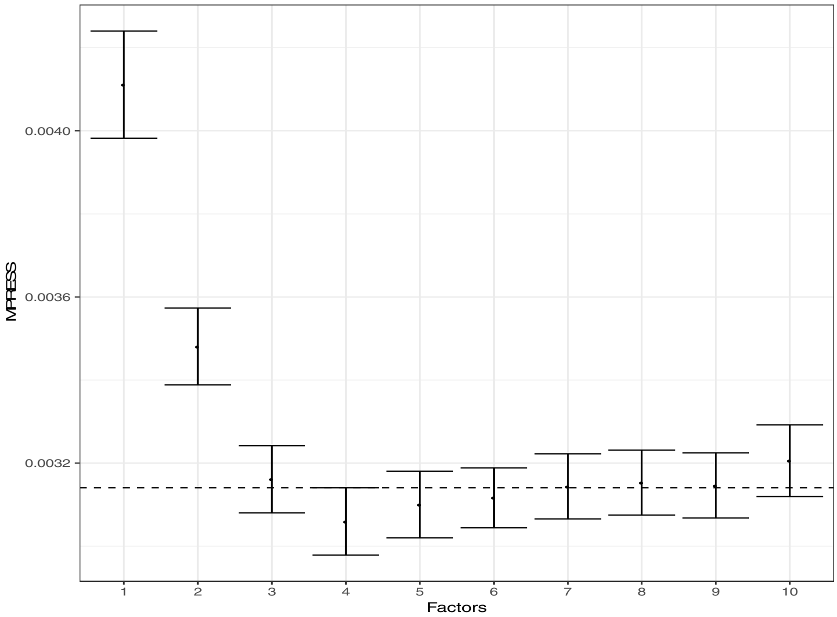

Figure 3. The MPRESS and standard error of the models with estimated with different numbers of components. The model estimated with four components was the model with the lowest MPRESS.

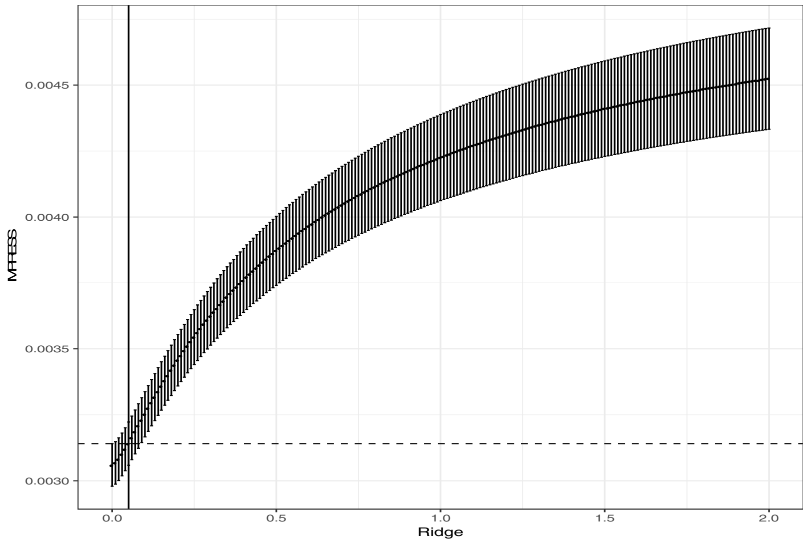

Figure 4. The MPRESS and standard error of the models estimated with four components for different values of the ridge parameter. The models below the dashed line are all models within one standard error of the model with the lowest MPRESS. The solid line indicates the ridge value that was picked for further analysis.

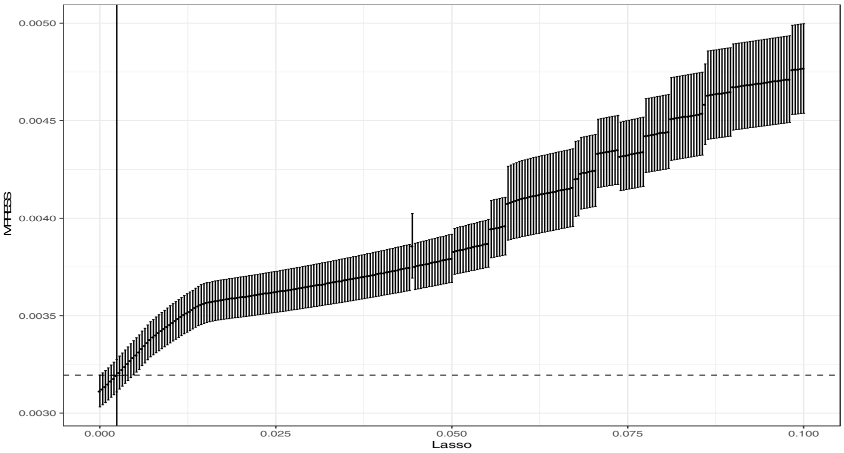

Figure 5. The MPRESS and standard error of the models for different values of the lasso parameter, with four components and the picked ridge tuning parameter. The models below the dashed line are all models within one standard error of the model with the lowest MPRESS. The solid line indicates the lasso value that was picked for further analysis.
